# 

**Published:** 1875-02-15

**Authors:** 


*ORIGINAL COMMUNICATIONS:*

Upon the Management of the Third Stage of Labor. By H. P. Merriman, M.D. ........ 87
A Bad Habit. By Charles Hervey Hunt, M.D. 94

*TRANSLATIONS:*

Progress of Medical Science in Germany. By E. J. Doering, M.D .......................... 96

*EDITORIAL DEPARTMENT:*

Mortality of Chicago in 1874. ................ 98

*CORRESPONDENCE:*

The Medical Schools and Hospitals of Paris.... 99

*SOCIETY REPORTS:*

Transactions of the Chicago Society of Physicians and Surgeons. Regular Meeting, Feb. 8, 1875 ........................................ 101
Minnesota State Medical Society........ ..... 105

*GLEANINGS FROM OUR EXCHANGES:*

The Therapeutics of Functional Headache. By Allan McLane Hamilton, M.D. ............ 106
Thoracentesis. .................................. 109
A Case of Thoracentesis. By Douglas Morton, M.D. ........................................ 111
The Action of Phosphorus as a Stimulant. By John Brunton, M.A., M.D. ............... 113

*BOOK REVIEWS:* .............................. 115

